# Factors Influencing the Evolution of Pulmonary Hypertension in Previously Healthy Subjects Recovering from a SARS-CoV-2 Infection

**DOI:** 10.3390/jcm10225272

**Published:** 2021-11-12

**Authors:** Cristina Tudoran, Mariana Tudoran, Voichita Elena Lazureanu, Adelina Raluca Marinescu, Talida Georgiana Cut, Cristian Oancea, Silvius Alexandru Pescariu, Gheorghe Nicusor Pop

**Affiliations:** 1 Department VII, Internal Medicine II, Discipline of Cardiology, University of Medicine and Pharmacy “Victor Babes” Timisoara, E. Murgu Square, Nr. 2, 300041 Timisoara, Romania; tudoran.cristina@umft.ro; 2 Center of Molecular Research in Nephrology and Vascular Disease, Faculty of Medicine, University of Medicine and Pharmacy “Victor Babes” Timisoara, E. Murgu Square, Nr. 2, 300041 Timisoara, Romania; 3 Cardiology Clinic, County Emergency Hospital “Pius Brinzeu”, L. Rebreanu Str., Nr. 156, 300723 Timisoara, Romania; 4 Department XIII, Discipline of Infectious Diseases, University of Medicine and Pharmacy “Victor Babes” Timisoara, E. Murgu Square, Nr. 2, 300041 Timisoara, Romania; lazureanu.voichita@umft.ro (V.E.L.); marinescu.adelina@umft.ro (A.R.M.); Talida.cut@gmail.com (T.G.C.); oancea@umft.ro (C.O.); 5 Department VI, Cardiology, University of Medicine and Pharmacy “Victor Babes” Timisoara, E. Murgu Square, Nr. 2, 300041 Timisoara, Romania; pescariu.alexandru@umft.ro (S.A.P.); pop.nicusor@umft.ro (G.N.P.)

**Keywords:** pulmonary hypertension, right ventricular dysfunction, transthoracic echocardiography, post-COVID-19 syndrome, long-term evolution

## Abstract

(1) Background: While the COVID-19 pandemic has been persisting for almost 2 years, more and more people are diagnosed with residual complications such as pulmonary hypertension (PH) and right ventricular dysfunction (RVD). This study aims to evaluate the course of PH and borderline PH (BPH) at 3 and 6 months after the acute COVID-19 infection and investigate if there are differences regarding its evolution between the patients from the first three waves of this disease. (2) Methods: We analyzed, by transthoracic echocardiography (TTE), the 3 and 6 months’ evolution of the echocardiographically estimated systolic pulmonary artery pressures (esPAP) in 116 patients already diagnosed with PH or BPH due to COVID-19 during the first three subsequent waves of COVID-19. (3) Results: We documented a gradual, statistically significant reduction in esPAP values, but also an improvement of the parameters characterizing RVD after 3 and 6 months (*p* < 0.001). This evolution was somewhat different between subjects infected with different viral strains and was related to the initial severity of the pulmonary injury and PH (adjusted R^2^ = 0.722, *p* < 0.001). (4) Conclusions: PH and RVD alleviate gradually during the recovery after COVID-19, but in some cases, they persist, suggesting the activation of pathophysiological mechanisms responsible for the self-propagation of PH.

## 1. Introduction

As the COVID-19 pandemic has been haunting for almost two years [1], causing the outburst of successive infection waves with constantly evolving strains of the severe acute respiratory syndrome coronavirus 2 (SARS-CoV-2), which are more virulent and pathogenic than the other [2,3], it has become obvious that this disease is associated with long-term complications and sequels of most organs and systems, with the respiratory and cardiovascular (CV) ones being the most frequent and well-known [4,5,6]. Terms as post-acute and long-COVID-19 have emerged to describe the prolonged persistence of symptoms and functional limitations, associated or not with objective pathological findings [7,8]. Whereas the acute COVID-19 infection often determines widespread lung injury, associated with diffuse involvement of the pulmonary vasculature, pulmonary microembolism or even thromboembolism, and myocardial lesions, the patients recovering from COVID-19 frequently accuse ongoing symptoms for a long time after the acute illness [9,10], justified in most cases by sequelae. One of these complications, which may explain some persisting symptoms such as dyspnea, fatigue, and reduced exercise tolerance, is pulmonary hypertension (PH) with a large spectrum of pathophysiological types, ranging from arterial PH (group 1), PH of group 3—due to lung disease and/or hypoxia—to chronic thromboembolic (group 4 PH) or even of group 2 PH (due to left heart disease) [9]. Whereas the prevalence of this pathology is a largely debated topic in the medical literature [11,12], there are little data concerning its outcome, and more emphasis has been put on the evolution of pulmonary lesions [13,14,15]. Although the diagnosis of PH would require right heart catheterism (RHC), the transthoracic echocardiography (TTE) allows an accurate estimation of the systolic pressure in the pulmonary artery (esPAP) and also offers an adequate evaluation of the right ventricular function (RVF), which is the most convenient method for the assessment and follow-up of these patients [16,17].

In this study, we aimed to investigate, by employing TTE, the evolution of esPAP and right ventricular dysfunction (RVD) at 3 and 6 months after acute SARS-CoV-2 infection in patients already diagnosed with PH and suffering from post-acute COVID syndrome, and to estimate the extent and timing of their improvement. Another aim was to investigate if there were differences regarding the course of PH between adult patients, without other significant associated pathologies, who suffered from a SARS-CoV-2 infection during the three waves of COVID-19.

## 2. Materials and Methods

### 2.1. Study Population

This study was conducted on a group of 116 patients already diagnosed with PH or borderline values of esPAP, associated or not with RVD, at 4 to 8 weeks after a SARS-CoV2 infection during the first [18], second [19], and third wave of COVID-19. They were identified from a total group of 383 subjects recovering from a SARS-CoV2 infection and who were referred to the outpatient cardiology or internal medicine services of our hospital for persisting symptoms such as dyspnea, fatigability, palpitations, chest pain/discomfort, and reduced exercise tolerance. As a consequence, they were all diagnosed with post-acute COVID-19 syndrome and were invited to attend a comprehensive cardiologic examination, including electrocardiogram (ECG) and TTE. Following these assessments, we diagnosed PH in 51 patients, associated or not with RVD, and in 65 other individuals, we assessed the borderline values of esPAP with or without borderline alteration of RVF. All these individuals were younger than 55 years and had no history of PH or significant pulmonary/CV diseases that could explain PH. They all underwent during the acute phase of the SARS-CoV2 infection an initial assessment consisting of chest computed tomography (CCT) and laboratory tests. Only subjects willing to attend a follow-up program were included in the study, namely a second CCT at 4–8 weeks after the acute pulmonary infection to document the regression of pulmonary injury, and cardiologic examinations with TTE at 3 and 6 months after the SARS-CoV2 infection, to follow the evolution of esPAP and/or of RVF.

Inclusion criteria:

1. Patients aged over 18 but younger than 55 years, to avoid the impact of age on pulmonary and CV structure and function;
2. The existence of a SARS-CoV2 infection within 4–8 weeks before the first exam, confirmed by a positive result of real-time reverse transcriptase-polymerase chain reaction (RT-PCR) assay of nasal and pharyngeal swabs, with a mild/moderate pulmonary injury during the acute phase, confirmed by CCT either during the hospitalization or as an outpatient during a COVID-19 evaluation, including CCT and laboratory tests;
3. The persistence of symptoms defining the post-acute COVID-19 syndrome;
4. An initial cardiologic examination with TTE, suggesting the presence of elevated esPAP values, with or without RVD, at 4–8 weeks after the acute infection and a second CCT assessment confirming the regression of pulmonary lesions;
5. The willingness to attend subsequent cardiologic examinations with TTE at 3 and 6 months after the COVID-19.

Exclusion criteria:

1. Patients under 18 or over 55 years old;
2. Individuals who did not agree to sign the informed consent or those not willing to undergo all assessments;
3. Subjects already diagnosed with PH before the COVID-19, those with a pre-existing history of other significant pulmonary and/or CV diseases, those who suffered confirmed pulmonary thromboembolism/deep vein thrombosis during COVID-19, or identified during the study with significant previously unknown cardiac pathology;
4. Patients without a pre-existing COVID-19 assessment, including laboratory tests and CCT describing the severity of their lung injury, and/or without a subsequent control confirming the regression of these lesions;
5. Patients with severe/critical forms of COVID-19 or with pulmonary infection associated with severe respiratory insufficiency, requiring mechanical ventilation.

### 2.2. Evaluation Methods

After signing informed consent, these patients were included in the study; their baseline clinical characteristics, CCTs results, laboratory data, as well as their pre-existing cardiologic examinations and TTE results, were collected from their medical records. The severity of the pulmonary injury was established based on the initial CCT performed either during the hospitalization or, in outpatients, on the COVID-19 evaluation together with laboratory determinations (especially C-reactive protein (CRP). According to these results, our patients were classified with mild (<30% pulmonary injury) or moderate (30–60% lesions) [20,21,22] forms. In order to evaluate their functional status, we assessed the severity of the post-acute COVID-19 syndrome and employed the Post-COVID-19 Functional Status (PCFS) scale. This is a methodology developed to estimate the severity of functional limitations during the recovery after COVID-19: 0 signifies “no limitations and symptoms”; 1—“negligible limitations of usual activities with persistent symptoms”; 2—“slight limitations with significant symptoms”; 3—“moderate limitations and not able to perform all usual activities due to symptoms, but still able to take care of himself without assistance”; 4—“severe limitation due to symptoms and requiring assistance to take care of themselves” [23]. 

Afterward, a comprehensive clinical exam, ECG, and TTE were performed in all subjects, which were repeated at 3 and 6 months after the acute infection. All TTE assessments were performed according to guidelines [16,17,24,25]. After a detailed examination of cardiac structure and function, we determined carefully several parameters characterizing RVT and allowing the estimation of esPAP:

1. Tricuspid Regurgitation Velocity (TRV) was determined from an apical window with continuous Doppler;
2. The estimated esPAP was assessed based on the peak TRV and adding the right atrial pressure (RAP), which was estimated by determining the inferior vena cava’s diameter, as well as its respiratory variations. In our study, we assumed that esPAP levels ≥35 mm Hg at rest indicates PH [24,26] with its gravity ranging from mild (35–44 mmHg) to moderate (45–60 mm Hg) and severe (>60 mm Hg) [25,26];
3. Tricuspid annular plane systolic excursion (TAPSE); was determined in M-Mode, at the lateral margin of the level of the tricuspid valve annulus; levels under 17 mm, were considered suggestive for RVD dysfunction;
4. Fractional area change (FAC) was determined in four chamber views; values <35% being considered pathological;
5. Right ventricular global longitudinal strain (RV-GLS) was determined in apical four chamber views [25,27];
6. The eccentricity index to differentiate RV volume and pressure overload by measuring two LV minor axes (one parallel to the interventricular septum and another perpendicular to it) at both end-diastole and end-systole.

According to the latest international recommendations, right ventricular dysfunction (RVD) was defined as either FAC < 35%, TAPSE < 17 mm, and/or RV-GLS < −28%, values placed in the close vicinity of these limits being considered borderline (TAPSE 17–20 mm; for FAC, between 35% and 36%; and for RV-GLS, −28 to −29) [24,25,27].

The Local Scientific Research Ethics Committee of our hospital approved the design and methodology of our study (No. 206/7.09.2020).

### 2.3. Statistical Methods

Numeric variables were presented as mean and standard deviation (SD) or median and interquartile range (IQR), and categorical variables were presented as frequency and percentages. We employed the Shapiro–Wilk test to check for the Gaussian distribution of numeric variables. The Chi-square test was used to evaluate the significance of differences in the proportions of clinical and laboratory findings. We employed the Mann–Whitney U test to compare general characteristics between patients with pulmonary hypertension and borderline. Kruskal-Wallis test was used to compare patients’ characteristics of the three waves. The evolution of TTE parameters and the PCFS scale over the three periods of time was analyzed by the Friedman test. For the evaluation of the potential connection between esPAP, RV-GLS, and other laboratory results, we employed Spearman’s correlation test. The individual impact of several confounding factors on the variance of continuous variables was assessed by building multivariate regression models. The quality of the model was described using the accuracy of prediction and R squared. The data analysis was performed using the Statistical Package for the Social Sciences v.26 (SPSS, Chicago, IL, USA). A *p*-value lower than 0.05 was considered statistically significant.

## 3. Results

This study was conducted on 116 patients who suffered from a SARS-CoV-2 infection confirmed by an RT-PCR test, with mild/moderate pulmonary injury (confirmed by a CCT) during the first, second, and third wave of COVID-19. They were all aged between 30 and 55 years, with a median of 48 (43–52.75) years. Fifty-seven of them were men (49.13%) and 59 women (50.86%). Their demographical, clinical, and laboratory characteristics are depicted in Table 1. They all underwent a cardiologic examination with a detailed TTE during 4–8 weeks after the acute illness, and were diagnosed with elevated values of esPAP, associated or not with altered RVF. In order to analyze the differences between the evolution of patients who suffered from COVID-19 during the different waves, we divided them into three subgroups: Group A—37 patients infected with the SARS-CoV-2 virus during the first wave; Group B—40 subjects infected during the second wave; Group C—39 patients diagnosed with COVID-19 during the third wave.

According to this criterion, 51 subjects had values of esPAP of over 35 mmHg, which placed them in the category of subjects with confirmed PH, associated with RVD or borderline RVF. In this subset, there were 28 men (54.9%) and 23 (45.09%) women, and their median age was 52 (45–54) years. They attended medical services accusing between two and four persisting symptoms and were framed according to the PCFS scale at a median level of 3. They all had a pulmonary injury on the CCT, most of them (76.47%) of moderate severity (Table 1). Using TTE, we determined increased esPAP values comprised between 36 and 55 mmHg, median 44.69 (40.52–48.56) mmHg. Forty-nine patients had associated RVD and two borderline values of FAC, TAPSE, and/or RV-GLS.

In the second subset, 65 subjects were included, diagnosed with esPAP values of under 35 but over 30 mmHg, which is not quite normal for their category of age. Twenty-nine (44.61%) were men, and 36 (55.38%) were women, and their median age was 46 (42.5–50) years, being somewhat younger than the first subset of patients. The majority of them claimed less persisting symptoms, considered at a median level 2, according to PCFS. They had a less severe pulmonary injury on CCT, namely mild forms in 80% of cases. Referring to their TTE examinations, they had esPAP values between 30 and 34.81 mmHg, which are higher than expected for this category of age. Concerning RVF, in 35 subjects (53.84%), we evidenced pathological values of one or more parameters defining RVD.

By analyzing these two subsets of patients, we observed that those with PH were older (*p* = 0.001), male gender prevailed, had higher BMI (*p* = 0.008) compared to patients with borderline values. As evaluated during the acute phase of COVID-19, patients with moderate forms of severity prevailed (76.47%, *p* < 0.001); based on the CCT, they had a more severe pulmonary injury and higher degrees of inflammation, with more increased CRP (*p* < 0.001). In the study, these patients accused more symptoms with higher PCFS levels (Table 1).

Referring to the TTE parameters, we noticed statistically significant correlations (*p* < 0.001) between the values of esPAP, determined both, initially, as well as during evolution, with the magnitude of the initial pulmonary injury assessed on CCT, with the initial CRP levels and the echocardiographic parameters characterizing RVF (TAPSE, FAC, and RV-GLS) (Table 2).

**Figure 1:**
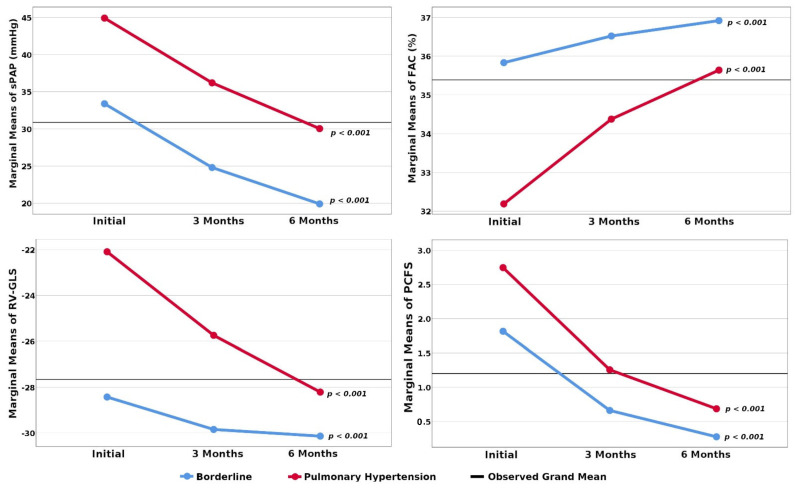
Evolution of patients’ characteristics at 3 and 6 months. Legend: esPAP—echocardigraphically estimated systolic pressure in the pulmonary artery; FAC—fractional area change; RV-GLS—right ventricular global longitudinal strain; PCFS—Post-COVID-19 Functional Status; *p*-statistical significance of Friedman test.

As we followed up on the evolution of these patients at 3 and 6 months, we noticed a gradual improvement in their clinical status and, especially in the TTE assessed parameters such as esPAP and those characterizing the RVF. Their progress, both in patients with PH and those with borderline values, is presented in Figure 1. At the end of the follow-up, from the total group of 116 patients, only 8 still had slightly elevated levels of esPAP (under 40 mmHg), and 17 had borderline values. Their RVF improved in parallel with the reduction in PH.

Starting from the premise that there could be some differences regarding the course of PH and RVD between the patients included in our study and who suffered from COVID-19 during different waves of this illness, we divided them accordingly and analyzed the results. Although our study comprises a small number of examined people (116), we noticed a higher prevalence of PH (15.57%, 20.3% borderlines) among the individuals from group C, who were infected mainly with the British strain of the SARS-CoV-2 virus during the third wave of COVID-19 in comparison to those affected during the first and second wave. As presented in Table 3, we observed some significant differences regarding the age of these patients (they were younger) and the severity of the pulmonary injury. Although the severity of PH and/or of RVD was higher in group C, the differences were not statistically significant when compared to the subjects from groups A and B.

**Figure 2:**
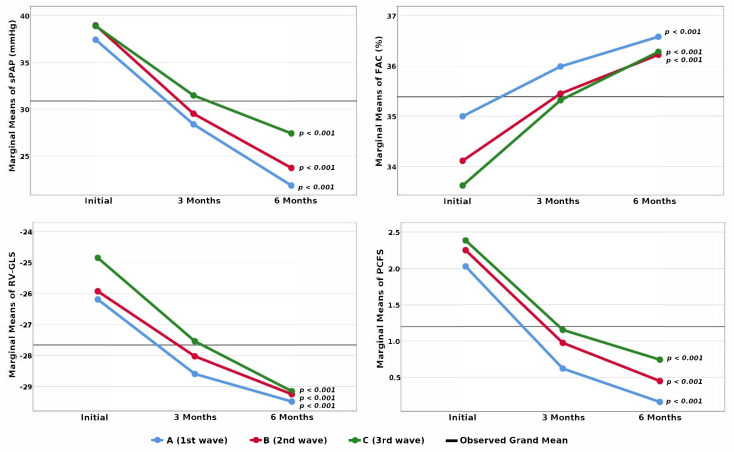
Evolution of esPAP, FAC, RV-GLS, and PCFS scale in patients with PH and borderline values who suffered from COVID-19 during the first, second, and third wave. Legend: esPAP—echocardiographically estimated systolic pressure in the pulmonary artery; FAC—fractional area change; RV-GLS—right ventricular global longitudinal strain; PCFS—Post-COVID-19 Functional Status; *p*-statistical significance of Friedman test.

The evolution of PH and RVD in all groups of patients was favorable, with a gradual reduction in esPAP and an improvement of the parameters characterizing RVF (Figure 2).

Starting from the premise that several factors could predict the evolution of PH, we used the multivariate linear regression analysis to build a regression model based on the forward stepwise method, and we employed the Akaike information criteria (AIC) to select the best model (Table 4). After the adjustment of several potentially confounding factors such as gender, age, and BMI, we identified the most significant predictors for the 6 months’ evolution of PH in our patient groups. Our results, obtained on relatively small populations infected with the SARS-CoV-2 virus during the first, second, and third wave of COVID-19, highlighted that the evolution of PH was somewhat different, probably due to the progressively increasing pathogeny of this agent. At 6 months, subjects from the second wave had, on average, esPAP 1.76 mmHg higher than those from the first wave, and, similarly, those from the third wave had increased mean values compared to those from the second wave. Other important elements were the magnitude of the pulmonary injury, assessed on the CCT during the acute infection, and the initial severity of PH, expressed by the levels of esPAP. Thus, an increase of 1% of the lung injury determined a higher mean value of esPAP at 6 months (0.127 mmHg), and an augmentation of the initial esPAP, with 1 mmHg predicted an elevation of 0.7 mmHg of the 6 months’ levels of esPAP. All these factors were responsible for 72.2% of the esPAP values at 6 months (adjusted R^2^ = 0.722)

## 4. Discussion

With the COVID-19 pandemic inducing a global health crisis, both through the burden on health services with the treatment of acute cases and through long-term care of the persisting consequences of this disease, recently, numerous papers discussing the complications of SARS-CoV-2 infection have been published in the medical literature [28,29]. Great importance has been given to the pulmonary and cardiovascular repercussions of this disease [11,18]. Of these, PH has aroused special interest, with many researchers focusing on the pathophysiology, epidemiology, treatment, and less on the long-term evolution of this complication [9,11,12]. Its prevalence varies largely depending on the studied population (elderly, patients from intensive care units, or younger outpatients), the severity of the COVID-19 form (mild, moderate, or severe) associated diseases, but generally, values of around 15% were advanced by Pagnesi et al. [11]. The prevalence of RVD is even higher, at around 39% [30], but exact data are lacking. That is why we only included younger patients in our study, where changes induced by aging on the lung and cardiovascular system are minimal, without pre-existent PH and heart diseases, or who suffered from a mild or moderate form of COVID-19 during the acute illness. After investigating 383 patients recovering from this disease, all who were diagnosed with post-acute COVID-19 syndrome, we established by using TTE, in our study group, a prevalence of around 13.3% of increased esPAP values, suggesting PH, but it varied from 12% and 12.66%, respectively, in patients infected during the first (the B1.1. subtype strain in most cases) and the second wave with south-east European strains (mostly the subtypes B1.5., B1.2., and B1.1.), to 15.7% in those from the third wave where the British strain of the SARS-CoV-2 virus (the strain VOC 202012/01) prevailed, although these were of younger age. Of course, compared to the enormous number of people infected with various strains of the SARS-CoV-2 virus, we are aware that our study group is too small to conclude statistically significant data.

According to guidelines [26], PH is suggested by TTE assessed esPAP of over 35 mmHg, but available data indicate that the normal esPAP values are lower; thus, the clinical significance of levels between 31 and 34 mmHg is unclear, and therefore, we considered them borderline; numerous subjects included in our study who fell in this category also highlighted aspects in other studies [30]. More than half of them had pathological values of the parameters characterizing RVF, raising the dilemma that these consequences of COVID-19, even subtle, could be much more frequent. These alterations could explain, at least partially, the persistence of symptoms such as dyspnea, fatigue, chest discomfort, and reduced exercise capacity for months after the recovery from the acute phase of COVID-19, sufferings described now by the term post-acute and long COVID-19 syndrome [7,28,31].

Several studies focused more on the regression of pulmonary lesions in these patients and observed residual abnormalities at more than 3 months after the acute illness, or even the development of interstitial lung disease [5,9,12,31,32]. Less attention was granted to the long-term outcome of PH, and that is why the principal purpose of our study was to follow, by TTE, the 3 and 6 months’ evolution of esPAP values, as well as of other TTE parameters characterizing RVF in patients already diagnosed with PH as a consequence of COVID-19. We observed a gradual improvement of these parameters after a follow-up of 3 and 6 months. Thus, after this period, we still noticed pathological values in several patients (15.68%).

It is, therefore, rational that patients recovering from COVID-19, especially those who had suffered from severe or moderate forms, should be followed up regularly to certify the regression of their pulmonary lesions and associated complications; some algorithms are even being proposed [10].

Study limitations: in this study, we did not certify our results, obtained by TTE, with the data of the RHC, and the natriuretic peptides were also not determined in all patients.

## 5. Conclusions

Elevated systolic pulmonary artery pressures suggesting pulmonary hypertension associated with right ventricular dysfunction are commonly encountered in patients recovering from COVID-19, explaining some of the persistent symptoms. They seem to improve gradually after 3 to 6 months from the initial infection; the extent of the recovery is related to the initial severity of this complication and of the lung injury, but apparently also to the pathogeny of the virus. In some individuals, pathological values persist, raising the suspicion either that it would take a longer time for the remission of these changes or that there are several pathophysiological mechanisms responsible for the self-propagation and maintenance of pulmonary hypertension.

## Figures and Tables

**Table 1:** Characteristics of the study population.

Patients’ Characteristics at the First Evaluation	51 Patients with PH	65 Patients with Borderline Values	*p*
Age (years)	52 (45–54)	46 (42.5–50)	0.001 ^a^
Gender: -male -female	28 (54.9%) 23 (45.09%)	29 (44.61%) 36 (55.38%)	0.271 ^b^
BMI (Kg/m^2^)	30.5 (27.47–31.80)	27.45 (25.09–30.31)	0.008 ^a^
Initial pulmonary injury on CCT: -Mild: ˂30% -Moderate: 30–60%	35 (31–40) 12 (23.52%) 39 (76.47%)	15 (6–27.50) 52 (80%) 13 (20%)	˂0.001 ^a^ ˂0.001 ^b^
Initial CRP (mg/dL)	43.57 (39.12–50.80)	30.28 (26.58–37.52)	˂0.001 ^a^
PCFS scale	3 (2–3)	2 (2–2)	˂0.001 ^a^
Echocardiographic parameters			
LAVI (mL/m^2^)	29.7 (21.59–34.85)	25.43 (18.46–33.39)	˂0.001 ^a^
LVEF (%)	43 (40–50)	55 (50–60)	˂0.001 ^a^
RA diameter (mm)	38 (37–40)	34 (33–35)	˂0.001 ^a^
RA area (cm^2^)	14.44 (13.69–16)	11.56 (10.89–12.25)	˂0.001 ^a^
RV diameter (mm)	33 (32–35)	28 (27–29)	˂0.001 ^a^
TRVmax: (m/s)	3.15 (2.98–3.30)	2.69 (2.61–2.70)	˂0.001 ^a^
esPAP (mmHg)	44.69 (40.52–48.56)	33.94 (32.24–34.16)	˂0.001 ^a^
TAPSE (mm)	16 (15–17)	20 (19–21.5)	˂0.001 ^a^
FAC (%)	33.11 (30.24–34.02)	35.85 (35.17–36.62)	˂0.001 ^a^
RV-GLS (%)	−22 (−25–−19)	−28 (−29–−28)	˂0.001 ^a^
EccI: End-diastole	1.4 (1.2–1.6)	1.1 (1–1.2)	˂0.001 ^a^
End-systole	1.4 (1.1–1.6)	1.1 (1–1.1)	˂0.001 ^a^

Legend: PH—pulmonary hypertension; *p*—statistical significance; BMI—body mass index; CCT—chest computed tomography; CRP—C reactive protein; PCFS—Post-COVID-19 Functional Status; LAVI—left atrial volume index; LVEF—left ventricular ejection fraction; RA—right atrium; RV—right ventricle; TRV max—maximum tricuspid regurgitation velocity; esPAP—echocardiographically estimated systolic pressure in pulmonary artery; TAPSE—tricuspid annular plane systolic excursion; FAC—fractional area change; RV-GLS—right ventricular global longitudinal strain; EccI—eccentricity index; ^a^—Mann–Whitney U test; ^b^—Chi-square test.

**Table 2:** Correlations between the initial and 6 months’ values of esPAP and other parameters.

Parameter	esPAP at the Initial Evaluation			esPAP after 6 Months		
	r	95%CI	*p*	r	95%CI	*p*
Initial pulmonary injury	0.821	0.736; 0.881	˂0.001	0.746	0.640; 0.815	˂0.001
Initial CRP	0.837	0.743; 0.899	˂0.001	0.725	0.602; 0.821	˂0.001
PCFS	0.713	0.604; 0.796	˂0.001	0.601	0.465; 0.705	˂0.001
TAPSE	−0.889	−0.929; −0.823	˂0.001	−0.782	−0.862; −0.671	˂0.001
FAC	−0.894	−0.929; −0.844	˂0.001	−0.745	−0.829; −0.633	˂0.001
RV-GLS	0.925	0.878; 0.957	˂0.001	0.783	0.680; 0.862	˂0.001

Legend: esPAP—echocardiographically estimated systolic pressure in the pulmonary artery; r—correlation coefficient; CI—confidence interval; *p*—statistical significance; CRP—C reactive protein; PCFS—Post-COVID-19 Functional Status; TAPSE—tricuspid annular plane systolic excursion; FAC—fractional area change; RV-GLS—right ventricular global longitudinal strain.

**Table 3:** Baseline characteristics of patients from the first, second, and third wave of COVID-19.

Patients’ Characteristics at the First Evaluation	Group A 37 Patients	Group B 40 Patients	Group C 39 Patients	*p*
Patients with PH ± RVD	15 (12% of 125)	19 (12.66% of 150)	17 (15.70% of 108)	0.826 ^b^
Borderline PH/RVD	22 (17.6% of 125)	21 (14% of 150)	22 (20.37% of 108)	
Age (years)	49 (45–54)	50 (46–53)	44 (40–47)	˂0.001 ^a^
Gender: male female	22 (59.45%) 15 (40.54%)	18 (45%) 22 (55%)	17 (43.58%) 22 (56.41%)	0.311 ^b^
BMI	27.72 (24.48–31.22)	30.12 (27.54–32.67)	27.73 (26.12–31.45)	0.067 ^a^
Initial pulmonary injury on TCT Mild: ˂30% Moderate: 30–60%	20 (5–35) 22 (59.45%) 15 (40.54%)	26.5 (15–35) 22 (55%) 18 (45%)	30 (25–38) 16 (41.02%) 23 (58.97%)	0.045 ^a^ 0.240 ^b^
Initial CRP	34.5 (25.62–41.95)	38.06 (30.30–43.29)	39.11 (29.67–41.82)	0.198 ^a^
PCFS	3 (2–3)	3 (2–3)	3 (2–3)	0.115 ^a^
Echocardiographic parameters				
TRVmax: PH ± RVD borderline PH/RVD	3.1 (2.95–3.29) 2.64 (2.60–2.70)	3.15 (2.97–3.30) 2.69 (2.64–2.70)	3.19 (3.03–3.40) 2.7 (2.60–2.71)	0.443 ^a^ 0.181 ^a^
esPAP: PH ± RVD borderline PH/RVD	44.18 (39.80–48.29) 33.3 (32.04–34.16)	44.69 (40.28–48.56) 33.94 (32.98–34.26)	46.73 (41.70–49.62) 34.16 (32.04–34.37)	0.443 ^a^ 0.181 ^a^
TAPSE: PH ± RVD borderline PH/RVD	16.63 (15.10–17.50)22 (20–22)	16 (13.67–17)20 (19–21)	15.37 (12.25–16.50)19 (18.75–20)	0.087 ^a^ 0.001 ^a^
FAC: PH ± RVD borderline PH/RVD	33.56 (31.57–34.56) 36.1 (35.59–37)	33.11 (30–34) 35.85 (35.10–36.61)	32.87 (29.89–33.67) 35.39 (34.78–35.89)	0.159 ^a^ 0.005 ^a^
RV-GLS: PH ± RVD borderline PH/RVD	−23 (−25–−19) −29 (−29–−28)	−22 (−26–−20) −29 (−29–−28)	−20 (−24–−17.50) −28 (−29–−27)	0.127 ^a^ 0.075 ^a^

Legend: Group A—patients recovered from the first COVID-19 wave with PH ± RVD/borderline cases; Group B—patients recovered from the second COVID-19 wave with PH ± RVD/borderline cases; Group C—patients recovered from the third COVID-19 wave with PH ± RVD/borderline cases; *p*-statistical significance; BMI—body mass index; CCT—chest computed tomography; CRP—C reactive protein; maximum tricuspid regurgitation velocity—TRV max; esPAP—echocardiographically estimated systolic pressure in pulmonary artery; tricuspid annular plane systolic excursion—TAPSE; right ventricular global longitudinal strain—RV-GLS; ^a^—Kruskall–Walllis test; ^b^—Chi-square test.

**Table 4:** Multivariate linear regression predicting the esPAP levels at 6 months.

Variable	β	Standard Error	*p*	95% CI for β
Wave	1.763	0.472	<0.001	0.827; 2.700
Initial Pulmonary Injury	0.127	0.046	0.007	0.036; 0.218
esPAP (initial)	0.702	0.084	<0.001	0.535; 0.868

Legend: esPAP—echocardiographically estimated systolic pressure in the pulmonary artery; CI—confidence interval; β—regression coefficient; *p*—statistical significance; CI—confidence interval.

## Data Availability

Our data are available at https://www.doi.org/10.17632/sbpc5gs85g.1/ last accessed on 9 November 2021.
